# Application of neural networks and neuro-fuzzy models in construction scheduling

**DOI:** 10.1038/s41598-023-35445-5

**Published:** 2023-05-21

**Authors:** Jude Iloabuchi Obianyo, Richard Chinenye Udeala, George Uwadiegwu Alaneme

**Affiliations:** 1 Department of Civil Engineering, Michael Okpara University of Agriculture, Umudike, P. M. B. 7267, Umuahia, 440109 Abia State Nigeria; 2 Department of Civil Engineering, Kampala International University, Kampala, Uganda

**Keywords:** Civil engineering, Engineering, Mathematics and computing

## Abstract

Construction scheduling is a complex process that involves a large number of variables, making it difficult to develop accurate and efficient schedules. Traditional scheduling techniques rely on manual analysis and intuition, which are prone to errors and often fail to account for all the variables involved. This results in project delays, cost overruns, and poor project performance. Artificial intelligence models have shown promise in improving construction scheduling accuracy by incorporating historical data, site-specific conditions, and other variables that traditional scheduling methods may not consider. In this research study, application of soft-computing techniques to evaluate construction schedule and control of project activities in order to achieve optimal performance in execution of building projects were carried out. Artificial neural network and neuro-fuzzy models were developed using data extracted from a residential two-storey reinforced concrete framed-structure construction schedule and project execution documents. The evaluation of project performance indicators in earned value analysis from 0 to 100% progress at 5% increment with a total of seventeen tasks were carried out using Microsoft Project software and data obtained from the computation were utilized for model development. Using input–output and curve-fitting (nftool) function in MATLAB, a 6-10-1 two-layer feed-forward network with tansig activation-function (AF) for the hidden neurons and linear AF output neurons was generated with Levenberg–Marquardt (Trainlm) training algorithm. Similarly, with the aid of ANFIS toolbox in MATLAB software, the training, testing and validation of the ANFIS model were carried out using hybrid optimization learning algorithm at 100 epochs and the Gaussian-membership-function (gaussmf). Loss-function parameters namely MAE, RMSE and R-values were taken as the performance evaluation criteria of the developed models. The generated statistical results indicates no significant difference between model-results and experimental values with MAE, RMSE, R^2^ of 1.9815, 2.256 and 99.9% respectively for ANFIS-model and MAE, RMSE, R^2^ of 2.146, 2.4095 and 99.998% respectively for the ANN-model. The model performance indicated that the ANFIS-model outclassed the ANN-model with their results satisfactory to deal with complex relationships between the model variables to produce accurate target response. The findings from this research study will improve the accuracy of construction scheduling, resulting in improved project performance and reduced costs.

## Introduction

Civil engineering construction and infrastructure development possess inherent constraints in vast areas, especially in the analysis, design and management of activities and interdependencies involved. The baseline for performing major decision-making processes is not only affected by several uncertainties, which are solved by deploying mathematics, mechanics and physics calculations, but also greatly depends on practitioners’ experience^1,2^. The knowledge gained in this process is ineffective and illogical in the absence of proactive precision, which cannot be properly executed when using a conventional computational approach to carry out multi-comparative statistical analysis^3^. Planning and scheduling of construction projects are inherently complex and involve accurate estimation of the number of project activities, their durations, sequence and amount of required resources, which is an area where artificial intelligence predictive modeling will be of significant support to generalize non-linear relationships between the project management constraints^4,5^. Moreover, the use of artificial intelligence (AI) in civil engineering projects has demonstrated limitless potential for creating smart, efficient management templates to improve decision-making precision and maximize cost and quality^6^.

Artificial intelligence is an area of computer science concerned with the study, design and use of intelligent computing, as well as processing complex data in a way that is inspired by the human brain. Artificial neural networks (ANNs) have the ability to learn and model non-linear relationships, which is really important because in real life, many of the relationships between inputs and outputs are non-linear as well complex^7^. The advantages of this modeling approach are high efficiency, continuous learning, wide applications, multitasking functioning and the ability to implicitly detect complex non-linear relationships between dependent and independent variables. In an attempt to emulate human cognition, neural networks are used today for a variety of reasons, including contractual relationships, fraud evasion, data retrieval, detection and surveillance. Neural networks are now thought of as common data-mining techniques and are used for a number of data-mining tasks, including pattern recognition, time series analysis, prediction and grouping^8,9^. However, an ANN can be a black box as it can approximate any function but cannot provide significant insights into the structure of the mathematical function being approximated. The combination of an ANN and fuzzy logic modeling approach can be referred to as neuro-fuzzy, also known as an adaptive neuro-fuzzy inference system (ANFIS). It helps to obtain suitable fuzzy inference classification through a learning hybridized optimization algorithm, to train the fuzzy system as well as derive appropriate membership function parameters of the fuzzy inference system such that the system models the complex input–output data. The neuro-fuzzy system also shares some advantages and characteristics with neural networks, such as its learning potential, assessment and optimization skills, and control systems. These facilitate the creation of a fuzzy-inference model from datasets using a unique learning approach motivated by learning metrics derived from neural networks^10^. Thanks to its capabilities concerning knowledge representation, automated learning and analysis of linguistic factors, the neuro-fuzzy model is a potent method for solving engineering difficulties and management issues for a given sophisticated system, such as prediction of decision support to improve the effectiveness of the reallocation and rescheduling processes^11,12^. The effectiveness of using a neural-learning technique implies that a fuzzy system with linguistic information in its rule base can be restructured or reformed using statistical data to produce a greater benefit than a neural network that is unable to use language information^13^. Fuzzy systems and neural networks have lately gained popularity as a combined method for handling control, identification, probability, and array appreciation problems in engineering domains. Recent years have seen an increase in artificial intelligence research, application implementation and tool creation^14,15^.

Similar to this, it is clear that AI is effective in the field of construction engineering and management, enabling users to achieve project objectives within budget and time constraints^16,17^. Yet, due to the complicated nature of many variable restrictions and the lack of clear or precise detailed information processing, the relevant research has shown that standalone AI systems have limits for handling non-trivial real-work situations^18–20^. Construction engineering and management constraints are classified according to their complexity, non-linearity, non-specificity, dynamism and uncertainty. For instance, fuzzy systems are particularly effective in evaluating the representation of explicit knowledge and making inferences^15,21–23^. Elmousalami^24^ investigated the appropriateness of computational intelligence techniques that included neuro-computing, fuzzy logic and evolutionary computation, which were modified for the evaluation of parametric cost-prediction models. Gregory et al.^25^ adapted a neuro-fuzzy soft computing technique for the prediction of the engineering performance in construction projects. Shahtaheri et al.^26^ proposed a predictive model based on an adaptive neuro-fuzzy inference system (ANFIS), employing 272 data points from 14 projects in the construction industry to approximate reference line tolls. Rashidi et al.^27^ used genetic and neuro-fuzzy systems to address the issue of choosing a skilled project manager. Similar to this, Shahhosseini and Sebt^28^ used an adaptive neuro-fuzzy inference system (ANFIS) to assign and select workers for construction projects depending on their qualifications.

The use of AI in the construction industry benefits both shareholders and investors in all phases of the construction process, including the proposal, costing and financing; material acquisition and correct execution; setup and resource management; and commercial prototype rehabilitation. In order to reduce the demand for experts in structure development and schedule designs, researchers and participants in construction-related projects develop technologies that resemble AI. To complete a project on time and under budget, a great project schedule is essential^29,30^. According to Schelle^31^, effective structure management entails the competent arrangement of several instances of contributing stakeholders, societies and fundamental building blocks. This might involve simulated elements such as tasks, errands and charges, as well as infinite associated units of diverging interactions. For building projects, when given step-by-step instructions and mandatory reinvigoration of responsibilities, this may allow complex undertakings to be managed successfully so that the intended results are achieved. Such recommendations and principles are given for large datasets that have existed over time and are perhaps active. They flow from one correctly specified form to the next properly outlined one. AlTabtabai^32^, for instance, employed a networked BP to launch a managerial method employing specialists chosen from the activities timetable, who supervised and predicted the repurposing of an abandoned many-story building.

This study uses the building of a residential, two-story, reinforced concrete framed structure in Nigeria as a case study to explore the application of artificial intelligence to construction scheduling in order to improve the project duration prediction and achieve cost minimization. Plus, we create an earned-value-management (EVM) model for better forecasting of the progress and performance and to enhance the efficiency of rescheduling and reallocation processes with the use of a decision support system by applying artificial neural networks and adaptive Neuro-fuzzy inference. A contractor’s bid and a construction timetable are equivalent. The timetable represents the estimated time necessary to complete the project, much like the bid represents the estimation of the cost that is assumed to be required to complete the project^33^. By using the building of a residential, two-story, reinforced concrete framed structure as a case study, this study aims to illustrate how artificial intelligence can be applied to construction scheduling in order to improve the project duration prediction and for cost minimization in Nigeria’s construction industry.

Additionally, earned-value-management (EVM) model is developed for better forecasting of the progress and performance and to enhance the efficiency of rescheduling and reallocation processes with the decision support system. A bid from contractors and a construction timetable are equivalent. Similar to how the bid is the estimate of the costs necessary to accomplish the project, the schedule denotes the anticipated amount of time needed to complete the project^34^. By using this, other stakeholders and general contractors can keep track of a project’s overall progress.

This study is aimed at applying artificial intelligence to construction scheduling to achieve better prediction of the project duration and minimize the costs in the building construction industry. The details derived from this research study will provide a new dynamic monitoring and optimization tool to track the progress of a project. The purpose of the research is to investigate the potential of neural networks and Neuro-fuzzy models in improving construction scheduling accuracy and efficiency and to provide insights into the application of these models in the broader field of construction engineering and management. A good construction project schedule is accurate, thorough and updated frequently, with communication regarding the project given first importance. Team cooperation is another important element since it helps tasks to be completed successfully. Scheduling allows project managers to match the labor, supplies, equipment and all other resources connected with activities and construction tasks over time, which is essential for the completion and success of a construction project. A well-planned construction schedule ensures the completion of projects by outlining the exact pace at which each job is to be completed, the sequences and methods for delivering resources, and the execution of all generated tasks^35,36^.

### Significance of the study

The application of neural networks and neuro-fuzzy models in construction scheduling is significant for several reasons. First, construction projects are complex and involve multiple tasks that need to be completed in a specific sequence. Any delay in one task can have a cascading effect on the rest of the project. Therefore, accurate scheduling is critical for the success of a construction project. Secondly, traditional scheduling methods rely on the experience and intuition of project managers, which can be subjective and lead to errors. The use of artificial intelligence (AI) models, such as neural networks and neuro-fuzzy models, can provide objective and data-driven scheduling solutions. Thirdly, the construction industry has been slow to adopt new technologies, and the application of AI in construction scheduling represents a step forward in the adoption of digital technologies. The use of AI models can help improve productivity, reduce project delays, and ultimately save costs. Overall, the significance of this study lies in its contribution to the development of more accurate and efficient scheduling methods for the construction industry, which can lead to improved project outcomes and better resource utilization.

### Project scheduling process

**Figure 1:**
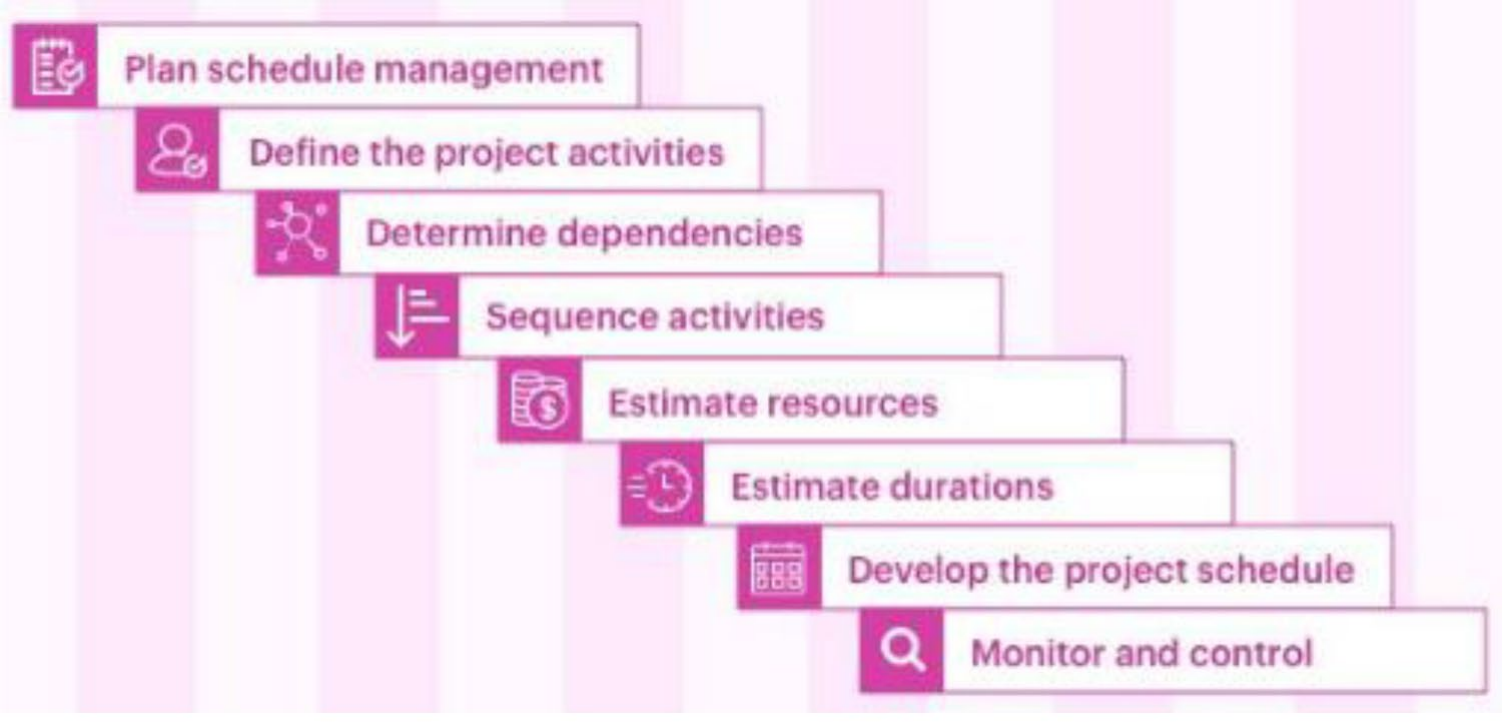
HYPERLINK "sps:id::fig1||locator::gr1||MediaObject::0" Project scheduling process.

The timetable for a construction project provides a clear view of all the project milestones, due dates and timelines. It should be regularly updated to measure the progress and show the various steps that must be taken before completion. The contractor’s ideas on how to complete the project are fully explained and demonstrated in a construction project schedule, which also clarifies the scope of the job. The necessary duration and the work activities are represented sequentially in the work scope. A project schedule is the only management document that can predict when a project will be completed, which is an important fact to be aware of. Scheduling involves the description of specific tasks and activities, as well as accomplishments that show a start date and an expected end date^37^. It is impossible to overstate how important scheduling is to a project’s success in construction. An effective timetable may be able to guarantee that the project is finished on time and within budget. It involves how and when a task is completed as well as how quickly the work is done. Furthermore, scheduling specifies the process and technique for material delivery. Finally, it allows for seasonal readjustments so that changes and uncertainties can be taken into account^28^. The task setup and timetabling rudiments can be divided into eight practicable stages, which enables timely execution within the designed budget as illustrated in Fig. 1.

## Methodology

The research study was carried out to examine feedback from the building/construction industry on the applications, utilization and feasibility of artificial intelligence in construction project scheduling using the established tender document. A deductive methodology was employed because it was best suited to the problem characteristics, and a qualitative approach was taken due to the investigative nature of the study. The study commenced with us conducting an in-depth literature review of relevant and recent scheduling methodologies used in the construction industry, with an emphasis on identifying their benefits and limitations^38^. The broad categories considered were design, procurement and execution. The information derived from the tender document serves as the foundation for periodic work valuation, variation valuation, variance reconciliation and all cost-related activities during building construction^39^. A project can be divided into several stages, with each representing a group of activities that culminate in the completion of one or more of the achievements, after usually having been completed in the order listed. This structuring divides the project into reasonable subdivisions for stress-free managing, designing and control. Depending on the nature of the project, each has different stages. The number of stages or the need for them is determined by several factors, including the project’s size, complexity and potential impact^40,41^.

**Figure 2:**
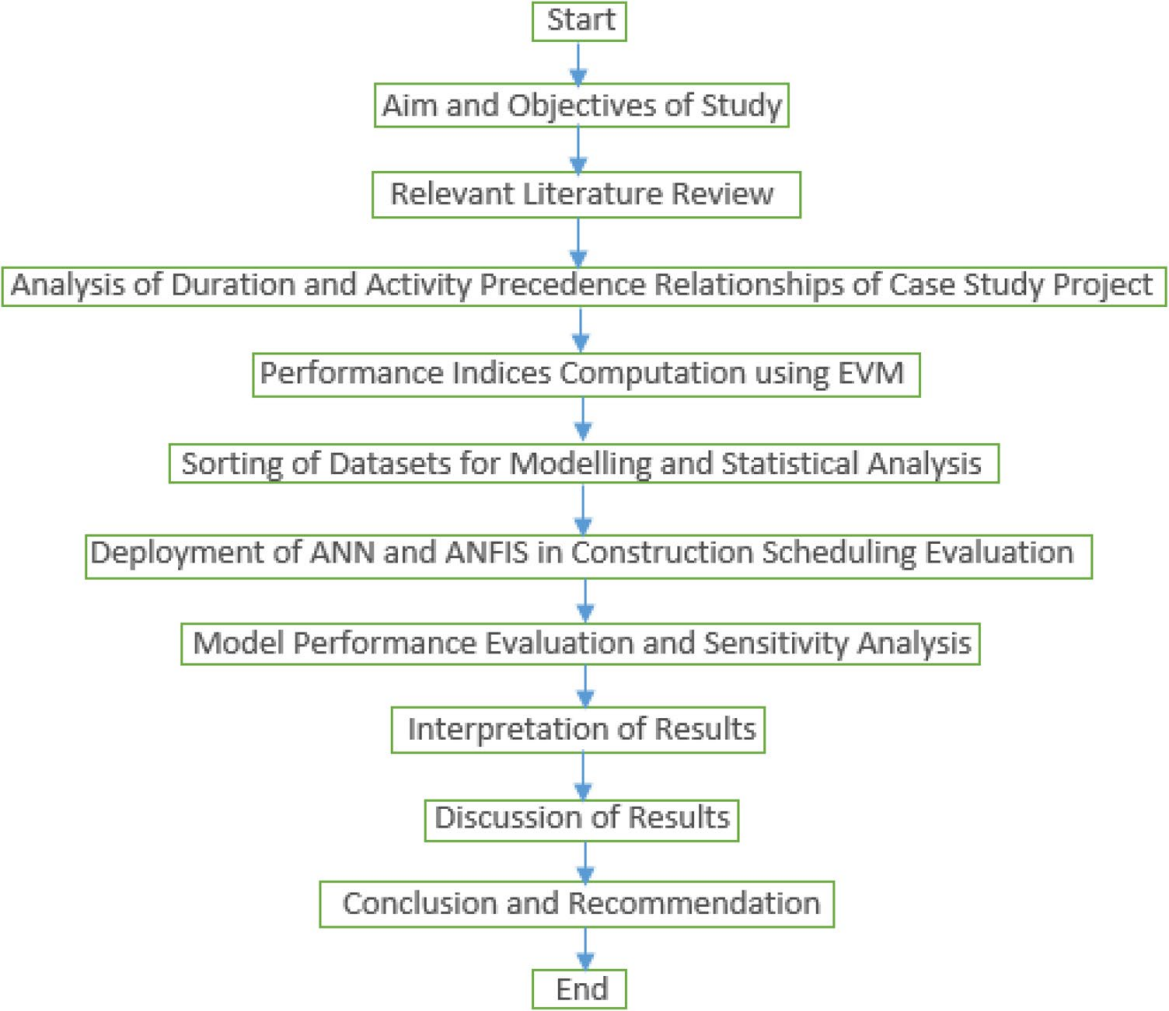
Research methodology flowchart.

Importantly, a tool for project management planning and analysis of the schedule is required given the relationships and interdependencies between the project’s activities. The critical path method, which is used in this research methodology, can be used to plan a large-scale activity network for project progress and management^42^. The start and end times of activities in the original schedule plan may be impacted, and the critical path may barely be reflected. Overcoming such issues, the critical path method is a project scheduling and analysis method that represents the tasks that must be completed in a specific project, including the trade-off between activity duration and cost. The basic rule is that any increase in critical activity duration leads to an increase in critical activity cost. The research methodology flowchart is shown in Fig. 2^43,44^.

### Steps to the critical path calculation

The critical path method (CPM) determines the task’s shortest achievable completion time using the project actions’ potential start and end times. In fact, more managers now view the critical path scheduling strategy as the most useful and practical scheduling technique. The duration denotes the shortest amount of time required to complete a certain project. If there is a barrier on the vital path, more time will be required before the project is completed. In order to use the critical path scheduling method in practice, construction task planners must act as a resource constraint via a precedence relationship^44,45^. The steps for calculating the CPM are stated below:

#### Forward-scrolling algorithm

This presents calculations for the critical path starting from the beginning of the node to the end of the grid, using Eq. (1)

1 $$ E(j) = \max \{ E(i) + D_{ij} \} $$

where *D*_*ij*_ is the activity duration, *E*(*i*) is the earliest start time for a given activity and *E*(*j*) is the latest start time^46^.

#### Backward-scrolling algorithm

This is the opposite of the front-scrolling algorithm. It calculates from the last node of the activity network and returns to the foremost node using mathematical relationships, as presented in Eq. (2)^47^.

2 $$ L(j) = \min \{ L(i) - D_{ij} \} $$

where *L*(*j*) is the latest end time for a given activity and *L*(*i*) is the earliest end time for a given activity. The difference (time) between the early start and the late start is known as elasticity, which represents the time in which the activity can be delayed without affecting the required project duration^40,48^.

**Figure 3:**
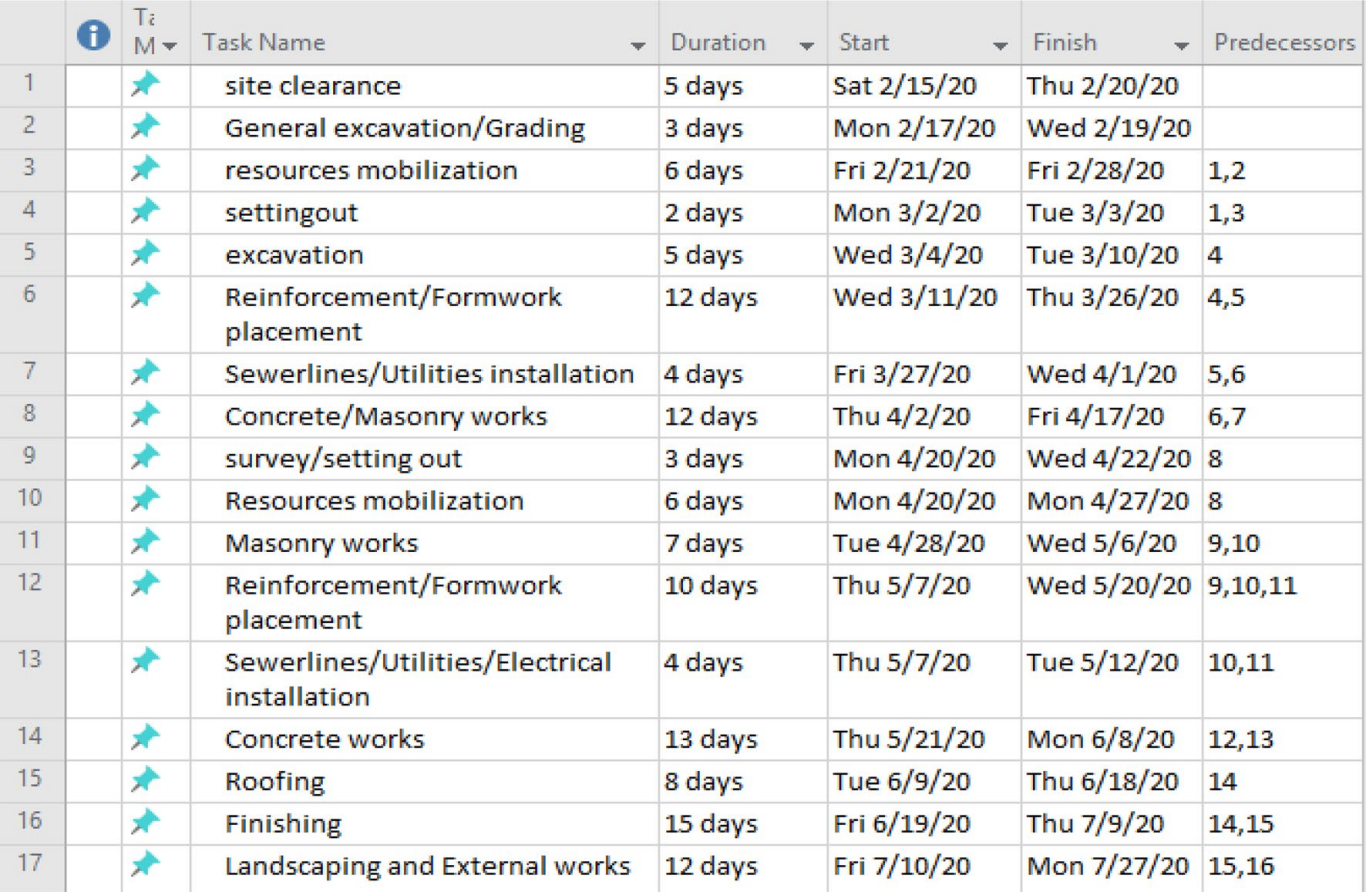
Precedence, relations and durations for a seventeen-activity project.

**Figure 4:**
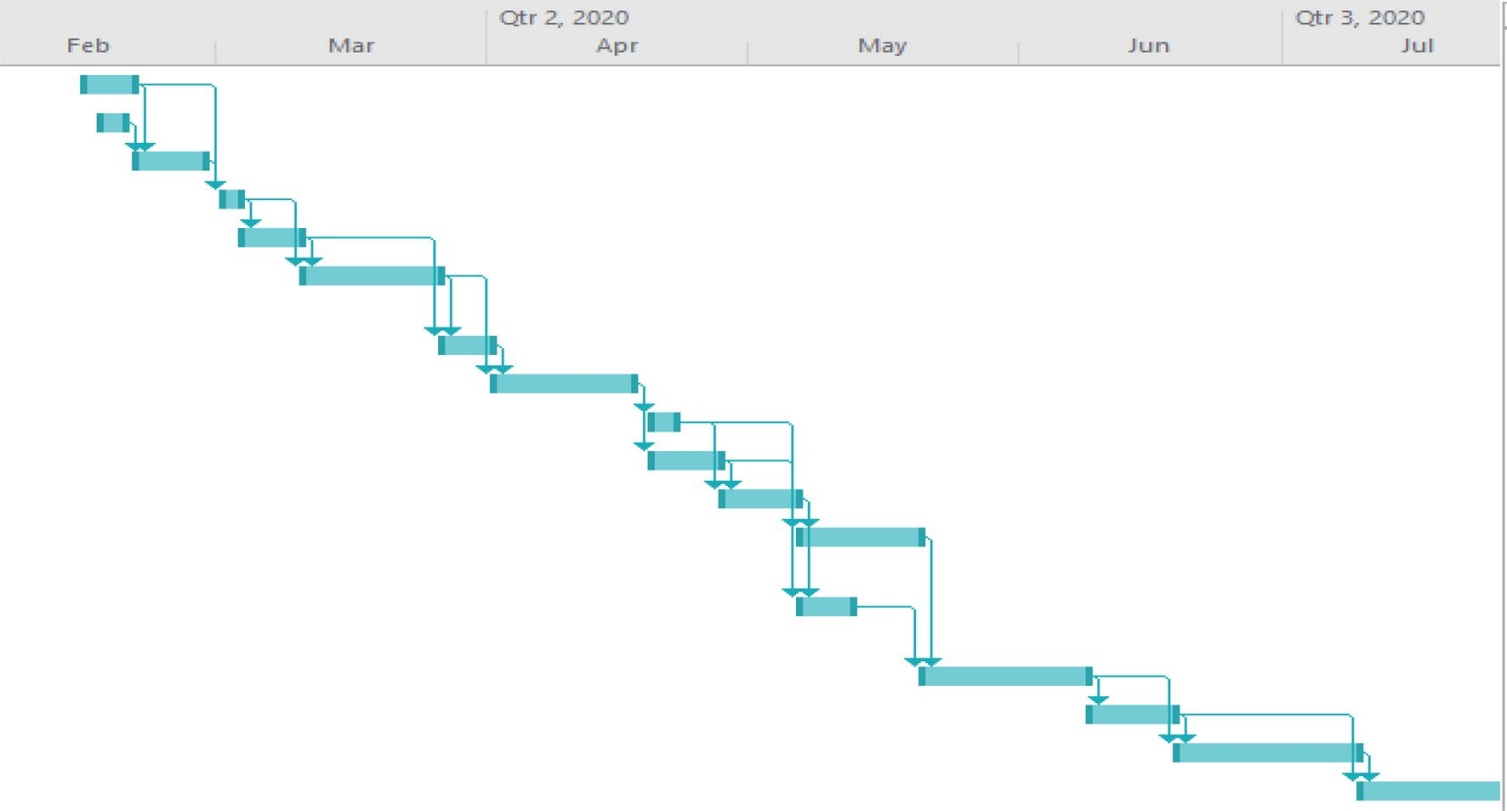
Gantt chart.

Using a two-story residential building project as a case study, the precedence, relations and durations for 17 activities required for the project are presented in Figs. 3 and 4.

### Calculating earned value

Earned value management (EVM) depicts in straightforward words the level of coverage and what tasks remain in a project. This accurate report is critical in recognizing faults, changing plans, amending mistakes and ensuring not only timely but also excellent delivery. The EVM puts cost and time on a unified scale, allowing one to graphically evaluate the actual work done vs. what was expected. The following direct indicators are adopted to appropriately scrutinize the timetable and costs accrued for a given mission using EVM^49^.

- Planned value (PV): is otherwise called the budgeted cost of work scheduled (BCWS). It is the cost sum through the current reporting period. It is the projected rate of a task arranged to conclude within an agreed interval^50^;
- Actual cost (AC): is also called the actual cost of work performance (ACWP). The actual cost implies the authentic payments made to complete a task by the set date. It is the recorded cost of completed works when using the preset interval alone;
- Earned value (EV): is otherwise referred to as the budgeted cost of work performance (BCWP). This is the aggregate task financial plan, increased by the percentage of task achievement. It denotes the accepted financial plan of tasks completed by the deadline^51^;
- Schedule Performance Index (SPI) and Schedule Variance (SV): the SPI is the ratio of EV to PV. It is a comparative quota of the project’s interval adeptness, which compares the actual headway to the premeditated headway. An SPI rate of < 1.0 designates that less work has been completed than anticipated, while a value of > 1.0 designates that more tasks were completed than were scheduled^35^. The SV is the variance flanked by the authentic tasks delivered contrary to the guesswork. It tells us whether the project is within plans or not. Zero variance depicts a project running according to the timetable/schedule, while a negative or positive difference depicts arrears or getting ahead of schedule, respectively^35^. The mathematical relationships are presented in Eqs. (3) and (4):
  3 $$ {\text{Schedule}}\;{\text{Performance}}\;{\text{Index}}\quad (SPI) = \frac{EV}{{PV}} $$
  4 $$ {\text{Schedule}}\;{\text{variance}}\quad (SV) = EV - PV $$
- Cost Performance Index (CPI) and Cost Variance (CV): the CPI is the ratio of EV to AC. It is a comparative quota of the cost of the project in terms of proficiency, which is capable of guesstimating the price of tasks left uncompleted^52^. The CV, therefore, stands for the variance between EV and AC. Whether a project is carried out as budgeted is showcased by the EV and AC. Zero indicates that the project is falling within the appropriated cost margins, whereas the project is considered as over or under the appropriate cost if the difference is negative or positive. The mathematical relationships are presented in Eqs. (5) and (6)^53,54^:
  5 $$ {\text{Cost}}\;{\text{performance}}\;{\text{index}}\quad (CPI) = \frac{EV}{{AC}} $$
  6 $$ {\text{Cost}}\;{\text{variance}}\quad (CV) = EV - AC $$

### Model performance evaluation

The performance of the intelligent model developed was evaluated in order to confirm that it has a proven ability to predict or estimate the target parameters with an acceptable degree of accuracy. Several performance criteria (statistical measures) used in the related literature, such as the loss function parameters, mean absolute error (MAE) and root mean square error (RMSE), are given with the formulas shown in Eqs. (7) and (8)^55–57^.

7 $$ RMSE = \sqrt {\frac{{\sum\nolimits_{i = 1}^{n} {\left( {E_{i} - M_{i} } \right)^{2} } }}{n}} $$

8 $$ MAE = \frac{1}{n}\sum\nolimits_{i = 1}^{n} {\left| {E_{i} - M_{i} } \right|} $$

where *n* is the size of the data points under investigation, *E*_*i*_ is the actual or experimental results and *M*_*i*_ is the estimated model values.

## Results, discussion and analysis

The schedule computation was carried out using Microsoft Project and Microsoft Excel software in line with the research carried out by Dayal^58^ for effective management of varying sizes of construction projects. The construction project under study was executed by a medium-sized firm with a planned duration of 95 days at an estimated direct cost of 25.8 million naira. The description of the project consisted of a residential, two-story, reinforced concrete framed structure with five bedrooms and a penthouse. The general information on the project was reviewed and the reasons for delaying the completion of the work. The critical and flexible activities involved in the project are presented in Table 1 from the computed results. The flow of the construction work’s 17 activities and dependencies indicated little or insignificant difference between the earliest and latest finish points of the project activities in the initial stages. However, as the project proceeded to the advanced stages, the relationships between the events and activities signaled appreciable slack periods, which provided necessary time for the safe completion of clashing preceding activities in the project^58,59^.

**Table 1:** Start and end times of the activities.

Task	Activity duration (days)	Earliest start	Earliest finish	Latest start	Latest finish	Slack
1	5	0	5	0	5	0
2	3	5	8	5	8	0
3	6	5	11	9	15	4
4	2	10	12	10	12	0
5	5	15	20	15	20	0
6	12	23	35	20	40	5
7	4	25	27	25	27	0
8	12	25	37	28	45	8
9	3	29	32	30	45	13
10	6	35	41	35	41	0
11	7	38	45	38	42	0
12	10	40	50	45	55	5
13	4	44	48	54	58	10
14	13	45	58	45	58	0
15	8	45	53	48	53	0
16	15	48	63	59	74	11
17	12	50	62	50	62	0

**Figure 5:**
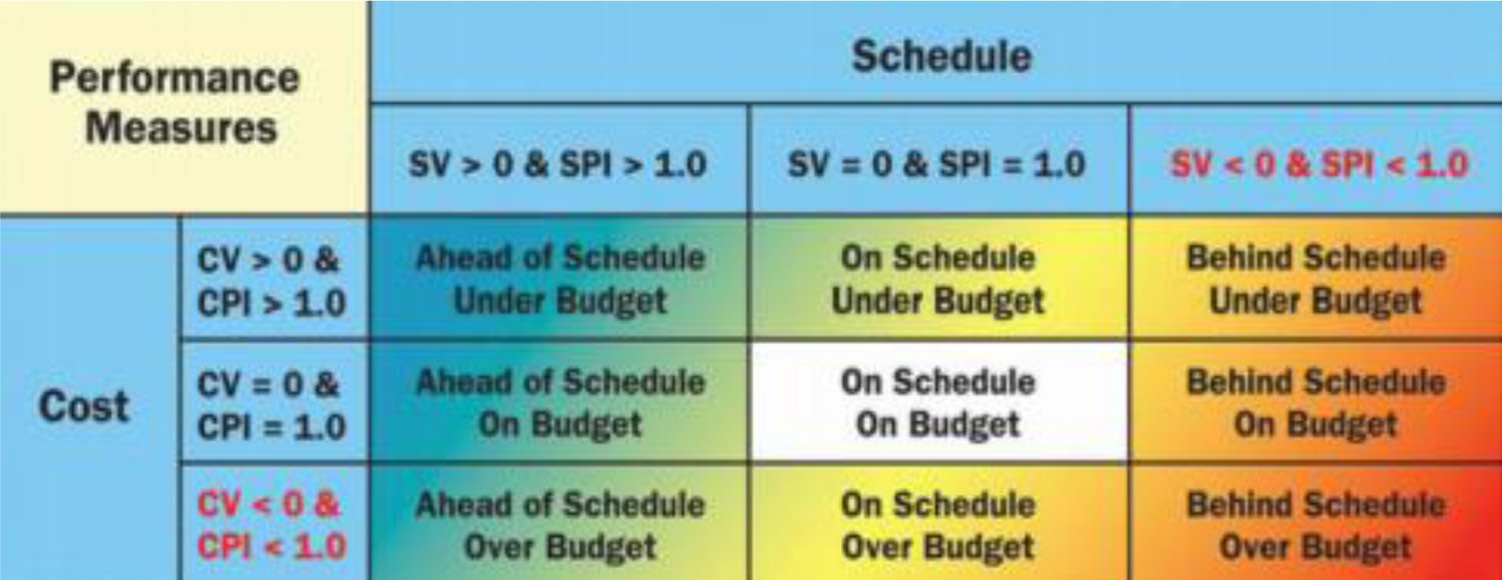
Interpretation of value for indicators of project performance (Kim et al.^53^).

The performance indicators’ computation results were extracted and are presented in Table 2, showing the actual time (AT), schedule variance (SV), earned value (EV), actual cost (AC), schedule performance indicator (SPI), cost variance (CV), cost planned progress and performance indicator (CPI) factors of the project. The interpretation of value for indicators of the project performance is shown in Fig. 5. The obtained results show a positive CPI and a CV observed to be > 1. These cost variables were further matched with the schedule computation outcome; the SPI was observed to be positive (> 1), and there was a negative SV (< 1)^35,60^. These derived results indicate that the project under study is behind schedule, and at the same time, under budget, as we can infer from the project performance interpretation chart. This occurred for several reasons, namely a lack of engagement of professionals to manage the project efficiently, along with environmental and safety factors. The obtained results are in agreement with the research study carried out by AnkurVerma^35^, who presented the significance, execution and distinctive elements of earned value management for promoting project success. Plus, the study carried out by Vanhoucke^50^ indicated the importance of calculating the indicators of the project performance in order to detect possible problems and identify solutions or mitigate constraints. The outcome of the project performance calculation further clarifies the need for the deployment of artificial intelligence techniques for the modeling of complex variables and constraints, to enable the smooth running of project activities through to their completion in the target time and within the estimated budget^61,62^.

**Table 2:** Performance indicators’ computation results.

Planned progress	AT (weeks)	ES	SV	SPI	EV	AC	CV	CPI
5%	2	2.22	0.22	1.11	646,800	638,560	8240	1.01
10%	3.5	3.75	0.25	1.07	687,200	671,790	15,410	1.02
15%	5	5.14	0.14	1.03	695,710	677,920	17,790	1.03
20%	6	6.28	0.28	1.05	728,350	710,500	17,850	1.03
25%	7.5	7.66	0.16	1.02	744,210	726,100	18,110	1.02
30%	8	8.35	0.35	1.04	789,820	771,580	18,240	1.02
35%	9.5	8.87	− 0.63	0.93	884,520	865,760	18,760	1.02
40%	10	9.31	− 0.69	0.93	988,470	971,280	17,190	1.02
45%	11.5	10.74	− 0.76	0.93	1,094,200	1,076,540	17,660	1.02
50%	13	12.58	− 0.42	0.97	1,197,245	1,178,150	19,095	1.02
55%	14	13.62	− 0.38	0.97	1,200,250	1,180,140	20,110	1.02
60%	15.5	14.89	− 0.61	0.96	1,504,560	1,478,190	26,370	1.02
65%	16	15.76	− 0.24	0.99	1,709,410	1,643,630	65,780	1.04
70%	17.5	17.33	− 0.17	0.99	2,510,560	2,442,240	68,320	1.03
75%	18	17.77	− 0.23	0.99	2,711,775	2,607,850	103,925	1.04
80%	19.5	18.61	− 0.89	0.95	3,612,350	3,378,590	233,760	1.07
85%	20	19.48	− 0.52	0.97	3,913,450	3,654,780	258,670	1.07
90%	21.5	20.85	− 0.65	0.97	5,014,025	4,687,870	326,155	1.07
95%	22	21.32	− 0.68	0.97	8,215,280	7,625,830	589,450	1.08
100%	23.5	22.54	− 0.96	0.96	10,718,470	10,058,920	659,550	1.07

### Datasets for model development

**Figure 6:**
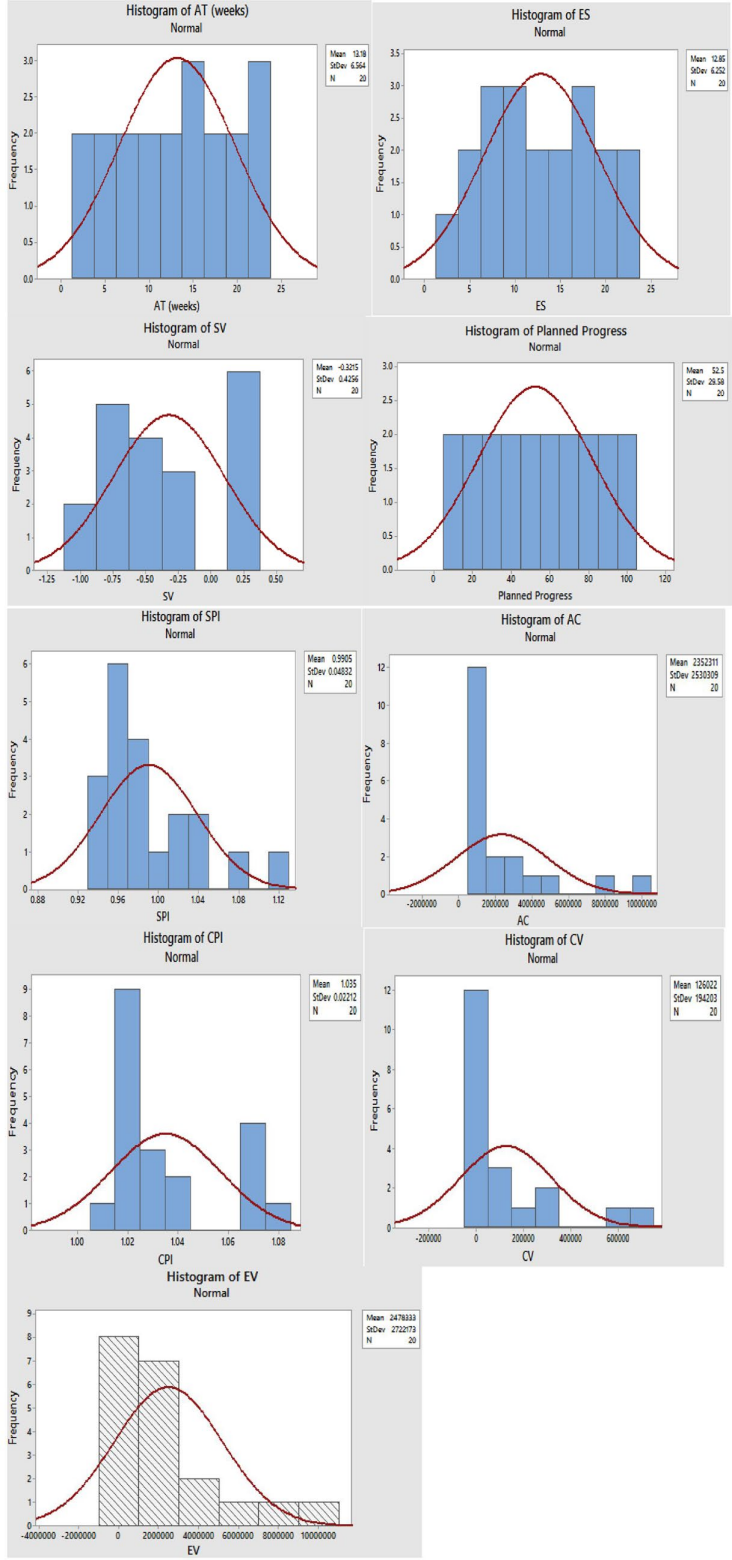
Distribution histogram chart for input and output variables.

Through expert judgment and consultations, the model variables were sorted to evaluate the performance indicators of the construction project. Distribution histograms were plotted for the model input and explanatory variables, as shown in Fig. 6, which present how often each value occurred in a dataset, showing slight or no skewness for the two parameters used^63^.

#### Pearson’s correlation

According to previous studies, Pearson’s correlation coefficients, as presented in Table 3, were deployed to evaluate the linear relationship between the predictors and explanatory variables. The results indicated strong positive relationships between the target response factor, earned value (EV), and the following performance indicators: planned progress, actual time (AT), earned schedule (ES), actual cost (AC) and cost variance (CV). Meanwhile, negative linear relationships were observed to exist for the schedule performance indicator and schedule variance factors^64,65^.

**Table 3:** Pearson’s correlations for model parameters.

	Planned progress	AT (weeks)	ES	SV	SPI	EV	AC	CV	CPI
Planned progress	1								
AT (weeks)	0.998535	1							
ES	0.997574	0.998976	1						
SV	− 0.74508	− 0.74708	− 0.71623	1					
SPI	− 0.63685	− 0.65127	− 0.62339	0.886424	1				
EV	0.801648	0.784082	0.783426	− 0.58361	− 0.33168	1			
AC	0.803279	0.785979	0.785319	− 0.58506	− 0.33414	0.999934	1		
CV	0.770749	0.749924	0.749324	− 0.55778	− 0.29563	0.988809	0.987036	1	
CPI	0.796671	0.779325	0.78482	− 0.48976	− 0.28396	0.819414	0.815283	0.863366	1

### Artificial neural network (ANN) model development

**Figure 7:**
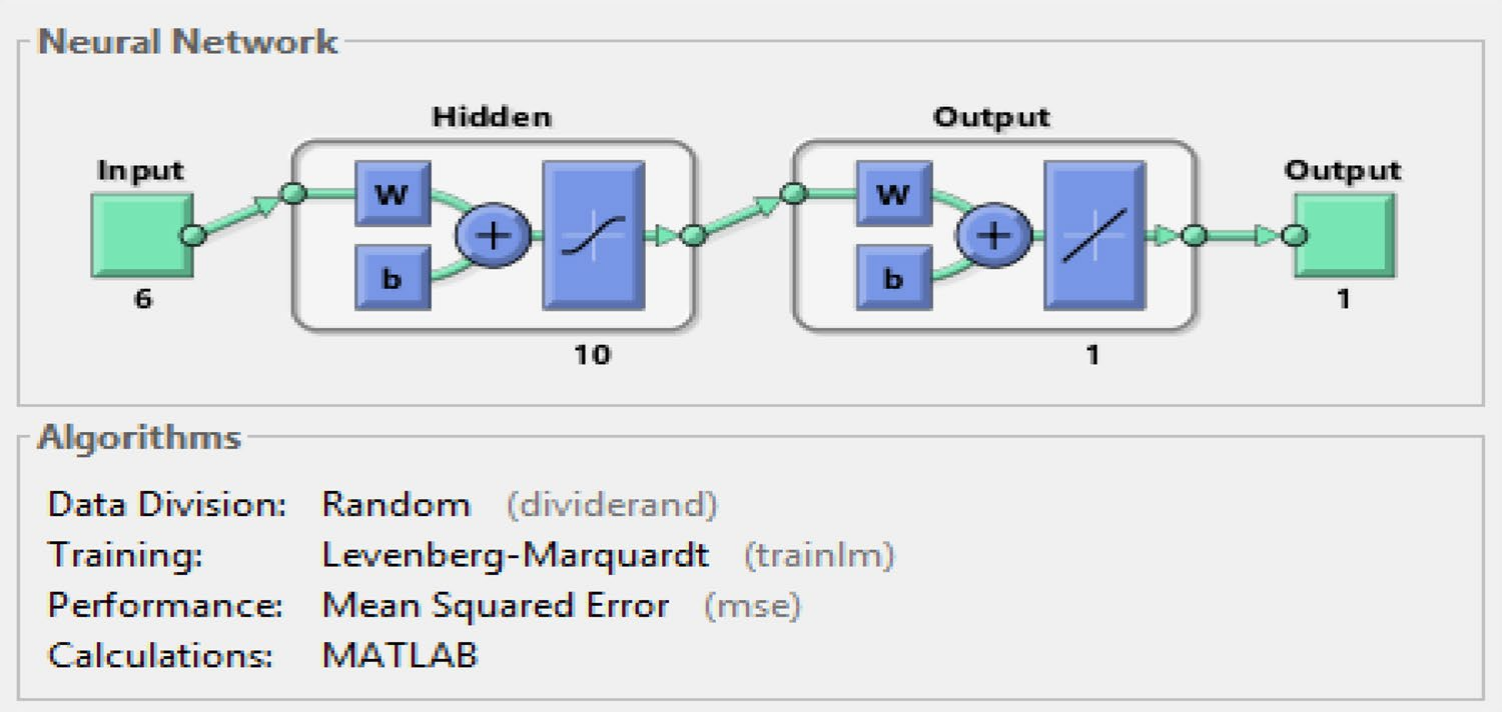
ANN architecture.

The modeling process was carried out with the datasets fed to the neural network using MATLAB software. The model framework was designed as six input variables namely, ES, planned progress, SV, SPI, CPI and AT; with one output parameter as the EV. The processing parameter settings for the neural network model are presented in Table 4 and Fig. 7, which show a 6-10-1 two-layer feed-forward network with a tansig activation function (AF) for the hidden neurons and linear AF output neurons. This can perform multidimensional mapping to solve complex system solutions. In order to determine the best-performing n-neurons, mean squared error (MSE) and R-values, evaluation criteria were used, which revealed that 10 neurons produced optimal results^66,67^.

**Table 4:** Artificial neural network processing parameter settings.

Parameters	Setting
General	
Type	Input–output and curve fitting (nftool)
Number of hidden neurons	10
Training function	Levenberg–Marquardt (Trainlm)
Data division	Random
Activation functions	Tansig, Purelin
Adaptation learning function	Gradient descent with momentum weight and bias learning function (Learngdm)
Performance	Mean squared error (MSE)
Calculation	MATLAB
Network type	Feed-forward backpropagation
System dataset sampling	
Training	70%
Testing	15%
Validation	15%

#### Training state of the ANN

**Figure 8:**
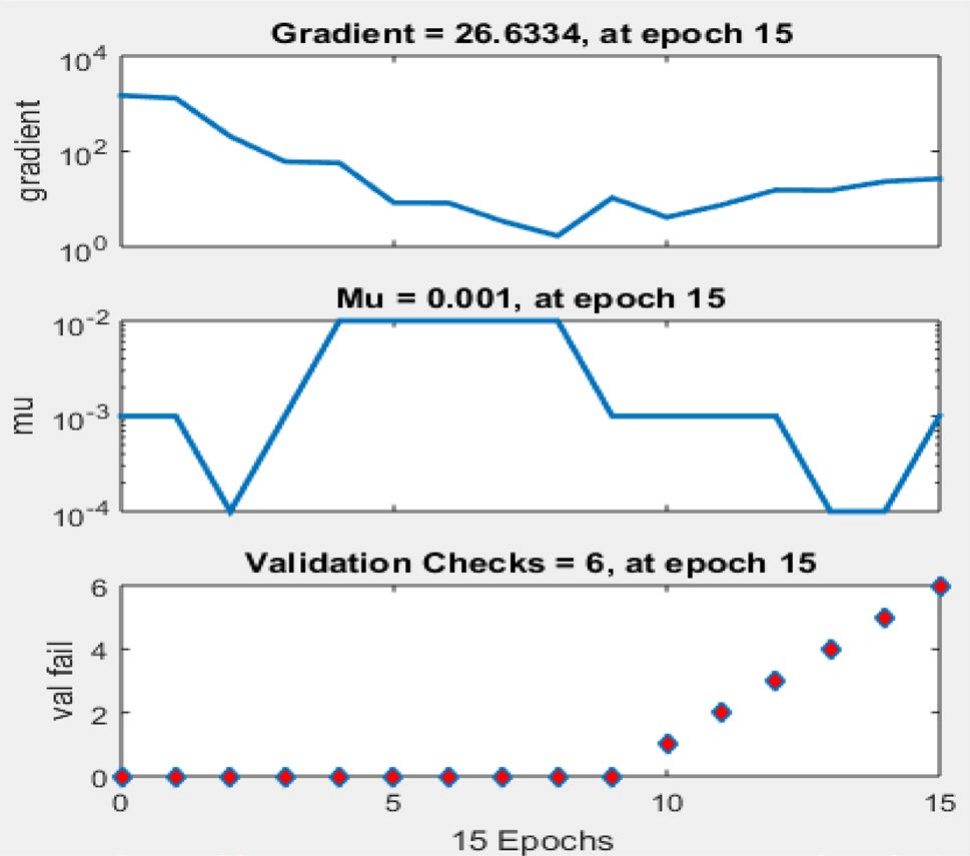
ANN training state.

The ANN training state plot (plottrainstate) of the neural network indicated a gradient of 26.6334, with the optimal value computed at 15 epochs. The validation checks failed at six because the errors were repeated six times before the process finally stopped. This represented the best performance of the neural network; at that stage, its performance ceased to improve further. The error function was repeated at zero points from epochs 0–9, then rose linearly from one to six over epochs 10–15. However, starting from epoch 10, we observed overfitting of the data. Therefore, epoch 9 was taken as the baseline, and its weight functions were selected as the final weights, as shown in Fig. 8^68^.

#### Validation performance of the ANN

**Figure 9:**
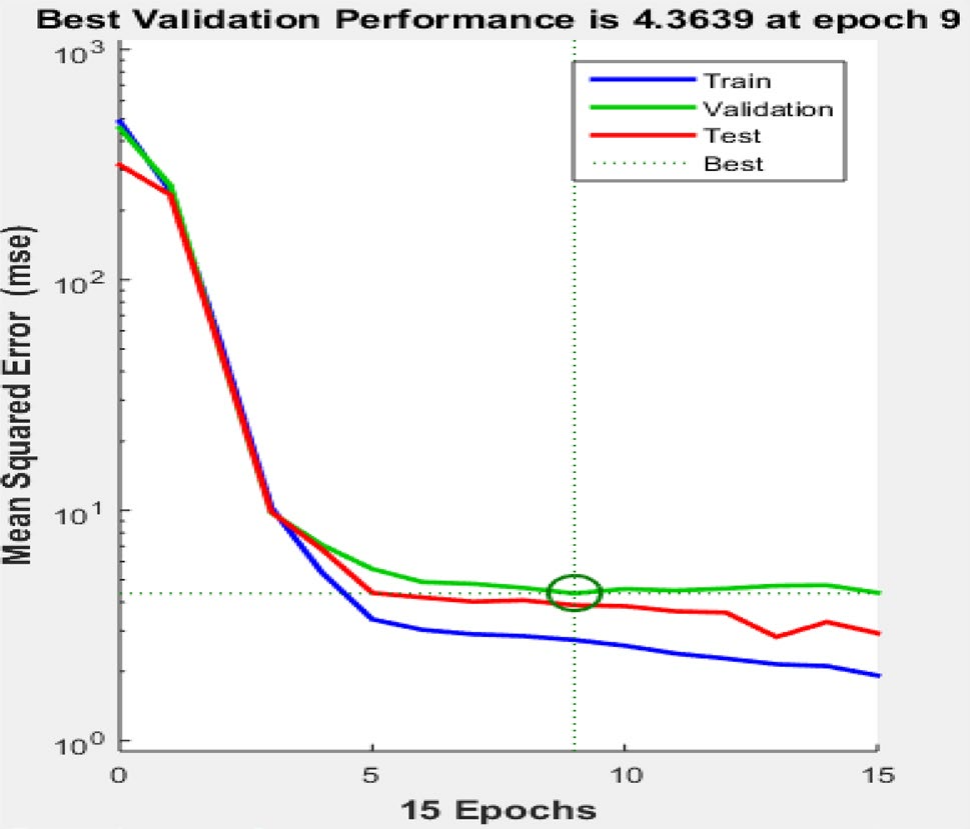
Validation performance of the ANN.

The mean square error (MSE) was the criteria tool used to evaluate the model’s performance while randomly selecting different hidden neuron numbers, activation function parameters and training algorithms for validation of the ANN network, as shown in Fig. 9. The graphical results indicated the best validation performance of 4.3639 at epoch 9 for the optimized network (8-10-1). The results indicated a satisfactory performance of the ANN model. It was capable of predicting the target response parameters accurately by generalizing the sets of complex input variables with minimum error^69,70^.

#### Error histogram of the ANN

**Figure 10:**
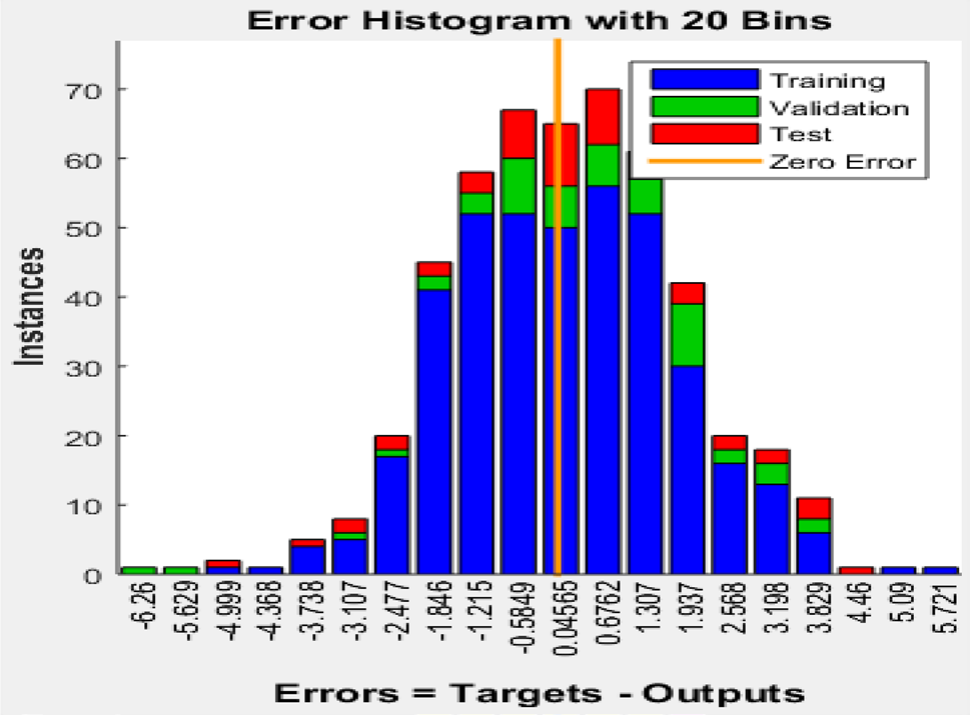
ANN error histogram.

An error histogram for the simulated smart model performance is presented in Fig. 10, which illustrates the level of correlation between the experimental and predicted variables with a 20 bins error histogram for training, testing and validation of the network. The zero-error point indicates the best performance during the simulation. Almost 95% of the data yield an error of less than 1%. The zero error is indicated with a yellow line in the middle at 0.04565 for the error function, with 50, 55 and 65 instances in the training, validation and testing sets, respectively^21,71^.

#### Regression plot of the ANN

**Figure 11:**
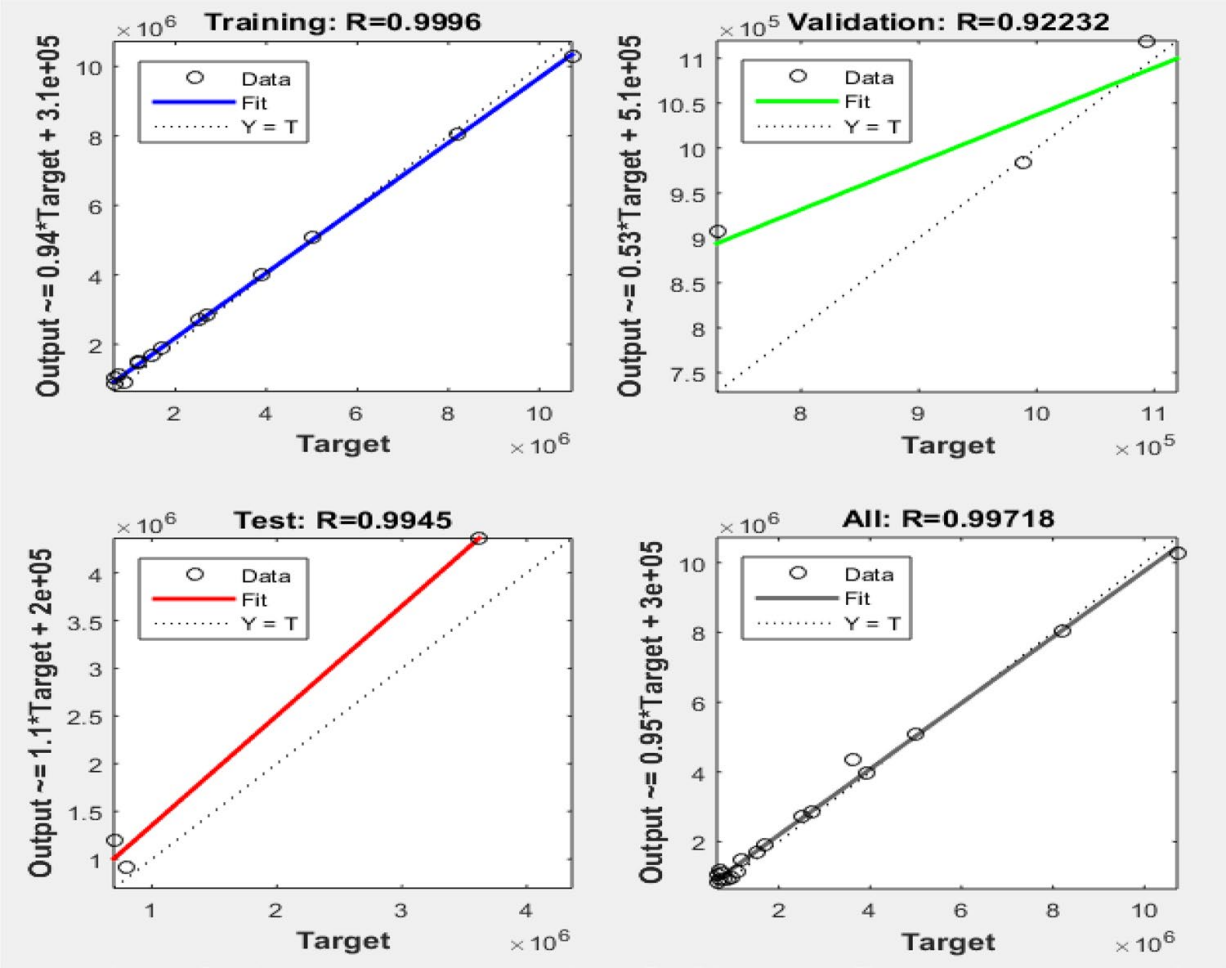
ANN training, testing and validation regression plots.

A regression plot presents the model relationships for the actual data and the ANN model results using the coefficient of determination and mean squared error (MSE) for the training, validation and testing sets, as shown in Fig. 11. The smart model output results were plotted on the *y*-axis of the regression plot while the actual values were on the *x*-axis. The derived statistical results show a satisfactory performance in terms of the prediction accuracy of the ANN model with 0.9996, 0.9945 and 0.92232 results obtained for training, testing and validation, respectively^72^.

#### Selection of the optimized ANN model

A comparison table showing various ANN architectures and their respective performance levels is shown in Table 5. The criteria used for performance evaluation of the network were the mean squared error (MSE), root mean squared error (RMSE) and coefficient of determination (r^2^). The optimized ANN model after network training and testing was the 6-10-1 architecture, with MSE, RMSE and r^2^ values of 4.639, 2.154 and 0.9994, respectively, for the training performance results. Similarly, the testing performance results for the optimized model were 2.354, 1.534 and 0.9945 for the MSE, RMSE and r^2^, respectively. The MATLAB script for the ANN model’s development and simulation, showing the connotation weight matrix, is presented in the attached supplementary file^73^.

**Table 5:** ANN architectures’ comparison to derive an optimized model during training and testing.

Model type	Architecture	Training			Testing		
		RMSE	MSE	r^2^	RMSE	MSE	r^2^
ANN-1	6-1-1	2.869495	8.234	0.99925	2.772724	7.688	0.9894
ANN-2	6-2-1	2.8265	7.9891	0.99918	2.930358	8.587	0.9824
ANN-3	6-3-1	2.706326	7.3242	0.99932	2.673387	7.147	0.9836
ANN-4	6-4-1	2.659887	7.075	0.99902	2.56203	6.564	0.9748
ANN-5	6-5-1	2.643047	6.9857	0.99898	2.612853	6.827	0.9779
ANN-6	6-6-1	2.596555	6.7421	0.99879	2.476288	6.132	0.9891
ANN-7	6-7-1	2.558613	6.5465	0.99888	2.290851	5.248	0.9902
ANN-8	6-8-1	2.47083	6.105	0.99917	2.377814	5.654	0.9921
ANN-9	6-9-1	2.378529	5.6574	0.99923	2.070266	4.286	0.9937
ANN-10	6-10-1	2.153834	4.639	0.99964	1.534275	2.354	0.9945
ANN-11	6-11-1	2.232062	4.9821	0.99877	1.791926	3.211	0.9928
ANN-12	6-12-1	2.290633	5.247	0.99892	1.89156	3.578	0.9913
ANN-13	6-13-1	2.25842	5.135	0.99899	1.86446	3.416	0.9924
ANN-14	6-14-1	2.38455	5.316	0.99888	1.89156	3.624	0.9833
ANN-15	6-15-1	2.44752	5.423	0.99878	1.89156	3.888	0.9805

### Neuro-fuzzy model development

**Figure 12:**
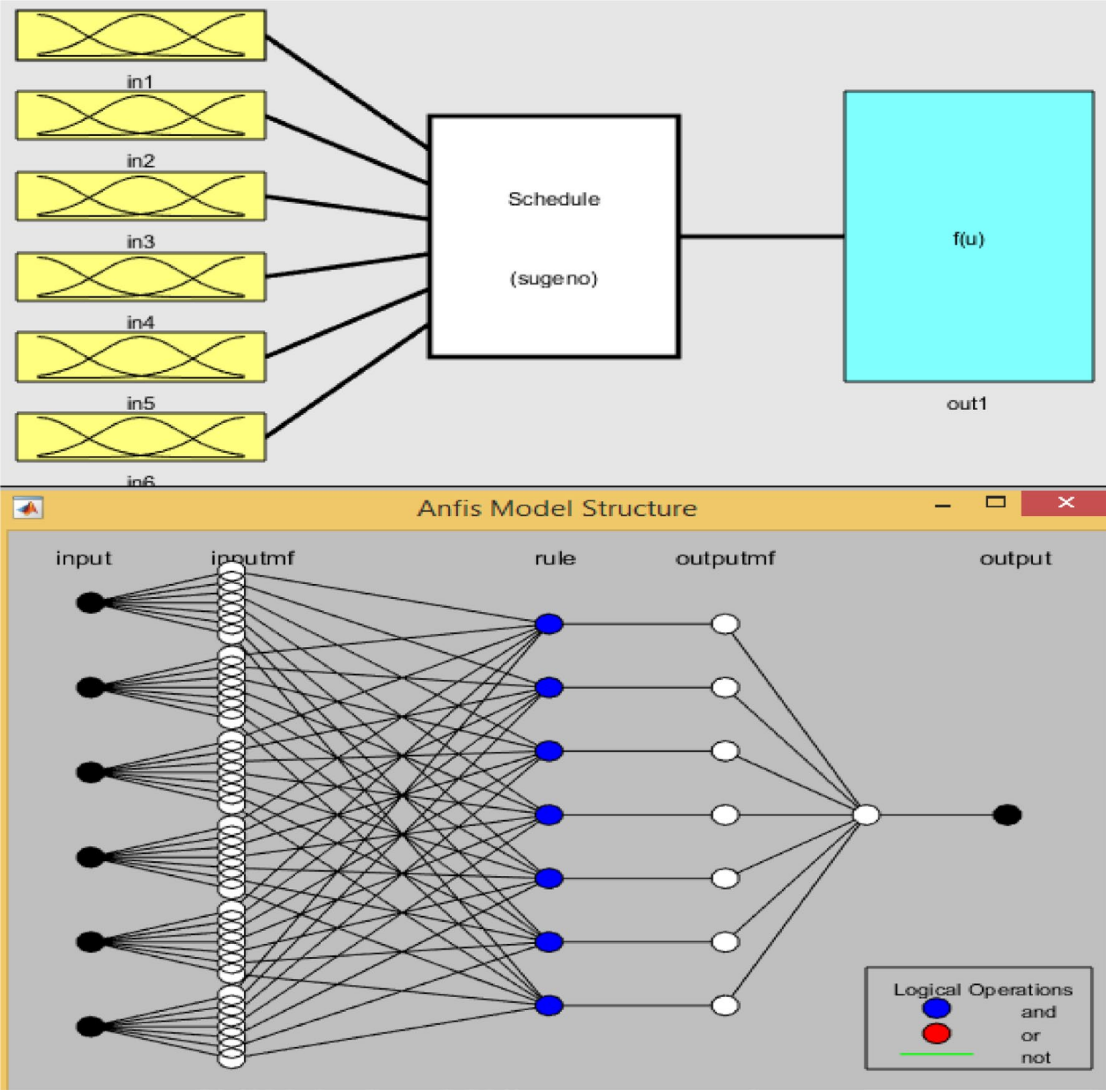
ANFIS model variables and architecture.

Detailed computation results showing the performance indicators of the cost and schedule for the project were utilized to build the ANFIS model input–output constraints appropriately. The earned schedule (ES), planned progress (percent), schedule variance (SV), schedule performance index (SPI), cost performance index (CPI) and actual time (AT) in weeks were the six input variables, and the earned value was the output variable (EV). The model variables’ relationships, showing the input–output associations, are given in Fig. 12^74^. The ANFIS model was trained, tested and validated using the ANFIS toolbox in MATLAB software. The MATLAB software workspace and membership function were generated using the sub-clustering fuzzy-inference-system formulation method after system datasets were loaded into it. Moreover, a hybrid method of optimization was deployed as the learning algorithm, which was adopted to train the fuzzy inference at 100 epochs^75^. Table 6 shows the learning and membership function constraints for data treatment, with an error tolerance value of 0, range of impact value of 0.5 and squash factor, reject and accept ratios of 1.25, 0.15 and 0.5, respectively. The Gaussian membership function (gaussmf) was utilized to evaluate the degrees of belongingness of the factors, as presented in Eq. (10). The model variables can be represented as follows:

$$ {\text{in1}} = {\text{ES in2}} = {\text{planned progress in3}} = {\text{SV in4}} = {\text{SPI in5}} = {\text{CPI in6}} = {\text{AT out1}} = {\text{EV}} $$

10 $$ f(x;\;\sigma ,\;c) = e^{{\frac{{ - (x - c)^{2} }}{{2\sigma^{2} }}}} $$

where $\sigma$ and $c$ represent the standard deviation and mean for the Gaussian function, respectively.

**Table 6:** ANFIS network parameters.

ANFIS network parameters	Settings
FIS type	Sub. clustering
Range of influence	0.5
Squash factor	1.25
Accept ratio	0.5
Reject ratio	0.15
Optimization method	Hybrid
Error tolerance	0
Epochs	100
Membership functions	7
Number of fuzzy rules	7
Membership functions type	gaussmf
Implication method	Minimum
Or method	Probor
And method	Prod
Aggregation	Maximum
Defuzzification	Wtaver

#### Testing and training ANFIS

**Figure 13:**
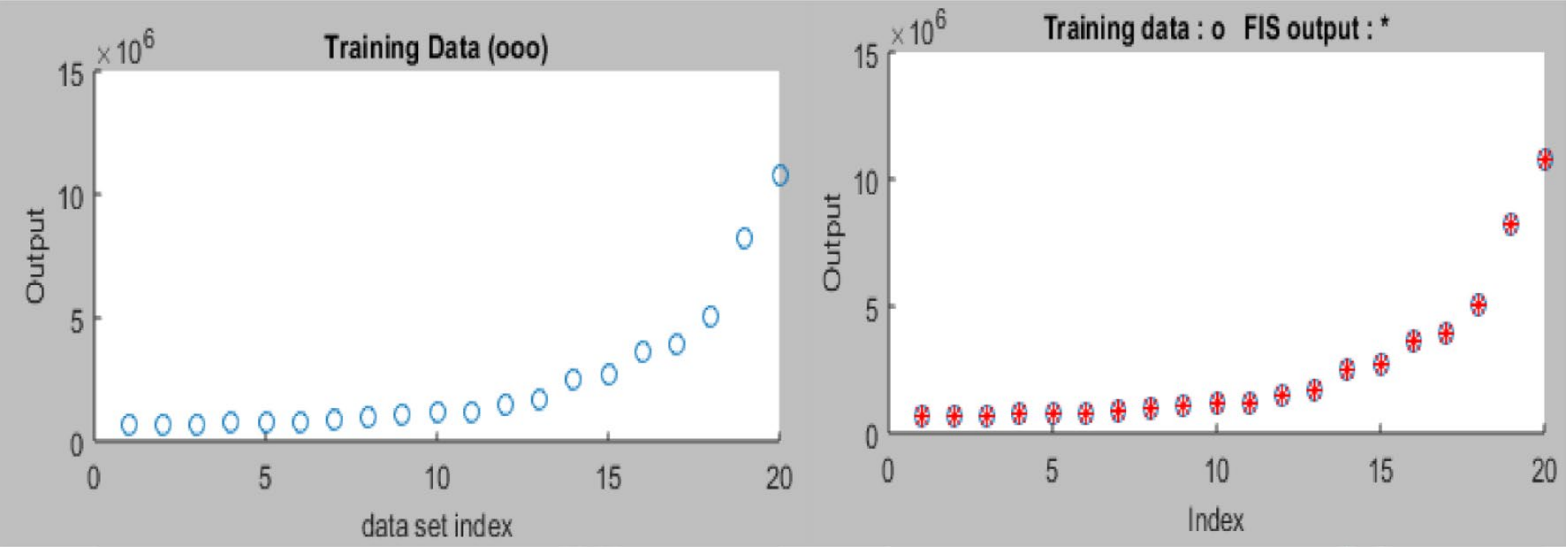
ANFIS model training and error plot.

**Figure 14:**
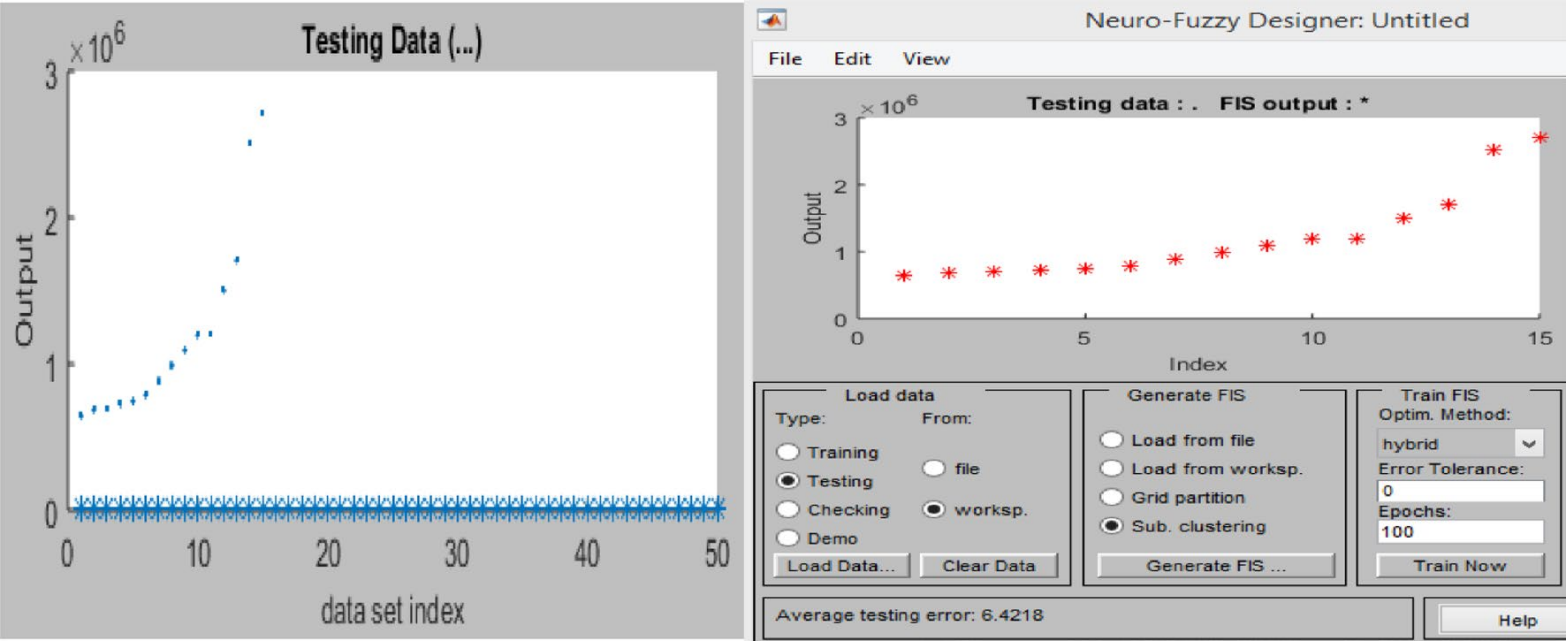
Plot of testing datasets.

To achieve the training, validation and testing of the neuro-fuzzy network using the prescribed hybrid optimization training methods and FIS constraints, the datasets used for the neuro-fuzzy modeling procedure were separated and arranged in two parts. The datasets were loaded from the workspace for ANFIS network training with one output and four input variables, as well as the graphical plot of 20 indices for network training. Training and testing error results of 8.0523 and 6.4218, respectively, were calculated in the process, as presented in Figs. 13 and 14^76^.

#### Graphical plots of the membership function

**Figure 15:**
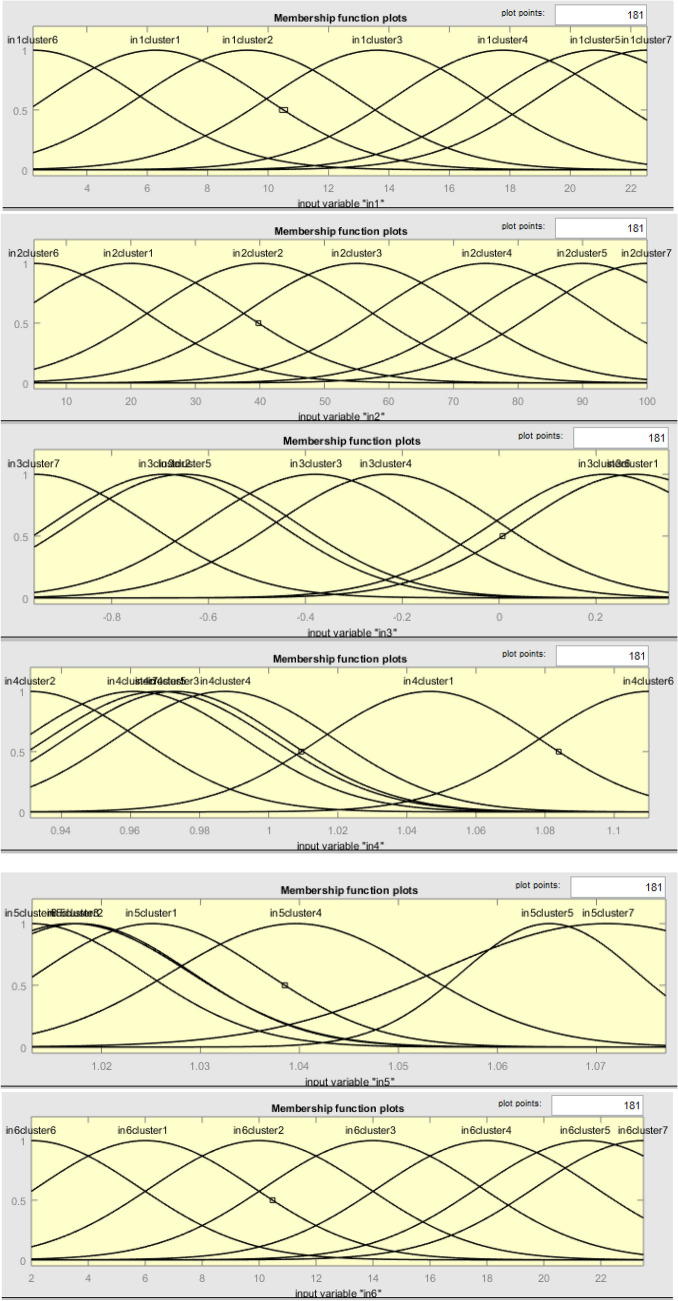
ANFIS membership function plots.

Graphical plots that show the membership function for the model variables in the ANFIS network were generated by means of the MATLAB recreation toolbox, which was used to robotically advance the suitable connection function standards to increase the records’ generality. Figure 15 shows the membership function designs, with the variety of records for model constraints on the *x*-axis and the discourse value from 0 to 1 on the *y*-axis^77^.

#### Selection of the optimized ANFIS model

A comparison table illustrating varying ANFIS network architectures and their respective performance using RMSE, MSE, and r^2^ is shown in Table 7. The optimized ANFIS model after network training and testing was the architecture type with a Gaussian membership function. The training performance results for the optimized model were 8.0523, 2.84 and 0.99999 for the MSE, RMSE and r^2^, respectively. Plus, for the testing performance, the optimized model produced values of 6.4218, 2.534 and 0.99999 for the MSE, RMSE and r^2^, respectively.

**Table 7:** ANFIS architecture comparison to derive the optimized model during training and testing.

Architecture	MF type	Training			Testing		
		RMSE	MSE	r^2^	RMSE	MSE	r^2^
ANFIS-01	trimf	3.201562	10.25	0.99895	3.158813	9.9781	0.99929
ANFIS-02	trapmf	3.512834	12.34	0.99771	3.356337	11.265	0.99918
ANFIS-03	gaussmf	2.837657	8.0523	0.99999	2.534127	6.4218	0.99999
ANFIS-04	gbellmf	3.445287	11.87	0.99689	3.226608	10.411	0.99935
ANFIS-05	psigmf	4.149699	17.22	0.99478	3.784574	14.323	0.99899
ANFIS-06	gauss2mf	3.053031	9.321	0.99924	2.952457	8.717	0.99911
ANFIS-07	pimf	2.983119	8.899	0.99966	2.761159	7.624	0.99957

#### ANFIS model variables’ graphical expression

**Figure 16:**
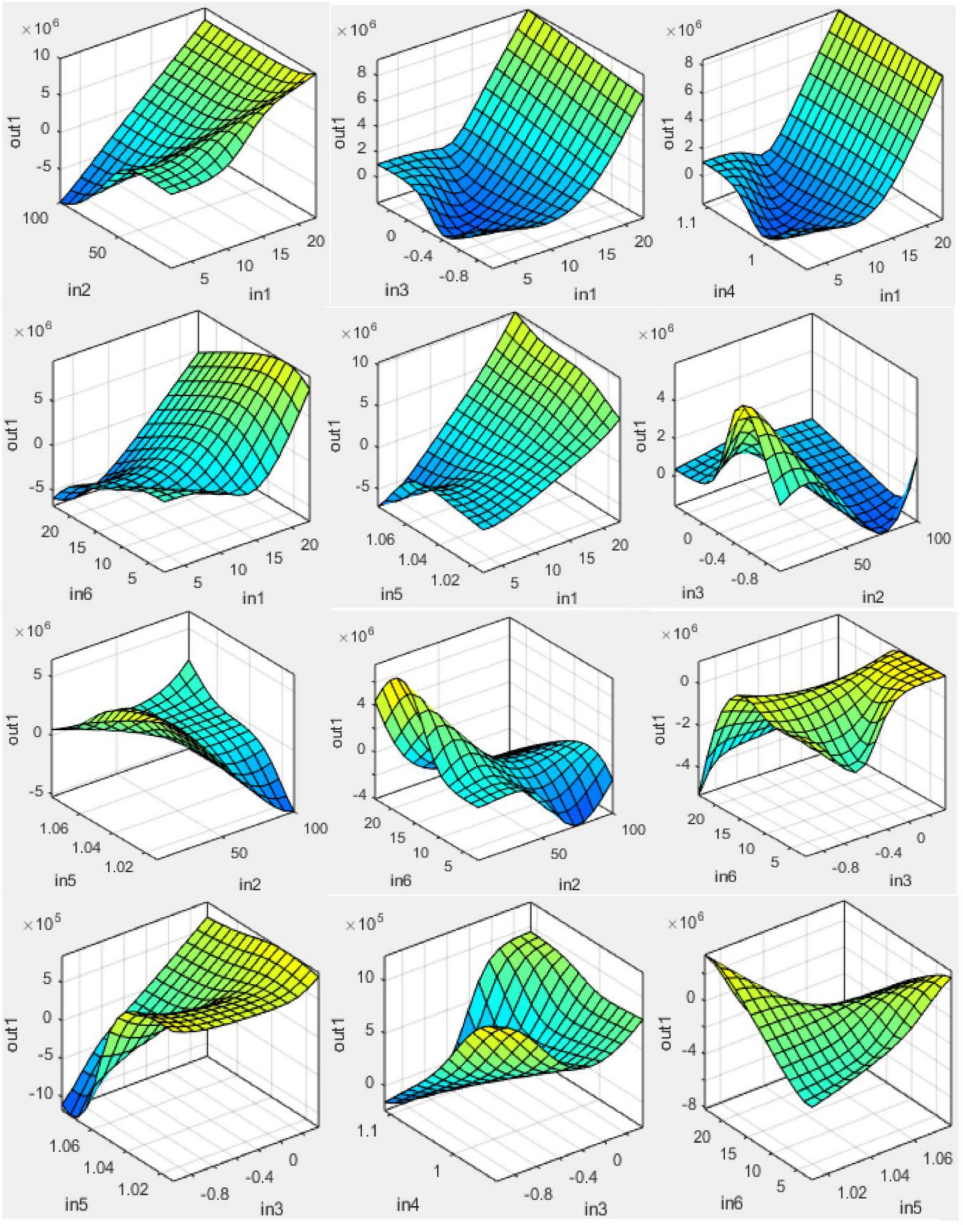
D-surface plots of ANFIS model variables.

A soft computing smart model was installed for the evaluation of the schedule performance indicators. This studies the generality of statistic sets it has been served with assistance from a hybrid optimization set of rules. Such a model has the power to precisely pair a given collection of inputs with the matching yield value. With a three-area apparent design, the prototypical variables’ interactions are weighed to spot their substantial one-to-one significance or possession, as revealed in Fig. 16. The influence of the independent variables on the earned value is assessed in this process^78^.

### Model validation

**Figure 17:**
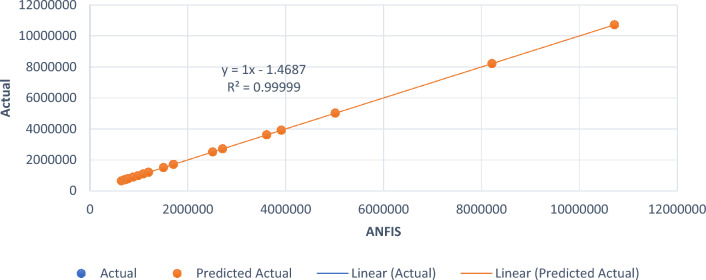
Goodness-of-fit plot for ANFIS model.

**Figure 18:**
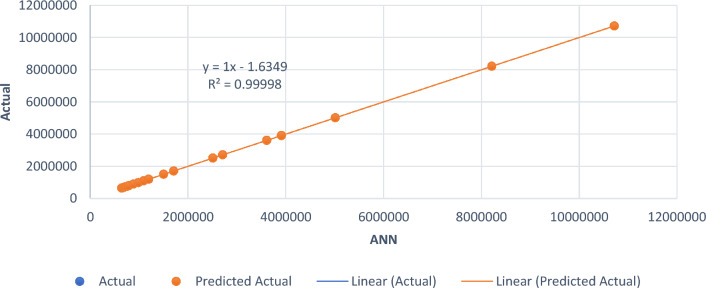
Goodness-of-fit plot for ANN model.

The developed smart intelligent model’s prediction performance was evaluated using a statistical method and loss function parameters, namely the mean absolute error (MAE) and root mean square error (RMSE). The evaluation was carried out for the ANN and ANFIS models. The model results and the actual values are presented in Table 8. The loss function statistical computation, which offered a good evaluation criterion for the performance of the developed smart intelligent model, is shown in Table 9. The generated statistical results indicate no significant difference between the model results and experimental values, with a MAE, RMSE and R^2^ of 1.9815, 2.256 and 99.9%, respectively, for the ANFIS model, and a MAE, RMSE and R^2^ of 2.146, 2.4095 and 99.998%, respectively, for the ANN model. Line-of-fit regression plots are shown in Figs. 17 and 18. The obtained statistical index results are in agreement with the findings of Alaneme et al.^56^ and Iro et al.^79^ for ANFIS and ANN model performance evaluation.

**Table 8:** Actual and model-predicted results.

Actual	ANFIS model	ANN model
646,800	646,802.2	646.801.7
687,200	687,201.5	687.202.4
695,710	695,713.21	695.714.56
728,350	728,351.11	728,348.7
744,210	744,210.2	744,211.5
789,820	789,820.5	789,818.6
884,520	884,521.13	884,524.13
988,470	988,472.4	988,468.4
1,094,200	1,094,203.2	1,094,202.3
1,197,245	1,197,244.82	1,197,248.95
1,200,250	1,200,249.2	1,200,253.42
1,504,560	1,504,559.36	1,504,558.36
1,709,410	1,709,410.14	1,709,411.02
2,510,560	2,510,560.33	2,510,559.02
2,711,775	2,711,774.88	2,711,776.64
3,612,350	3,612,349.22	3,612,348.47
3,913,450	3,913,452.14	3,913,448.15
5,014,025	5,014,024.44	5,014,026.19
8,215,280	8,215,281.06	8,215,279.46
10,718,470	10,718,470.43	10,718,469.73

**Table 9:** Performance evaluation of the developed model.

Target output	Statistical parameter	Requirements	Calculated results	Remarks
ANFIS model	MAE	Close to 0	1.9815	Excellent
	RMSE	Close to 0	2.257	Very good
	R^2^	Greater than 0.8	0.99999	Excellent
ANN model	MAE	Close to 0	2.146	Excellent
	RMSE	Close to 0	2.4095	Very good
	R^2^	Greater than 0.8	0.99998	Excellent

### Sensitivity analysis

**Figure 19:**
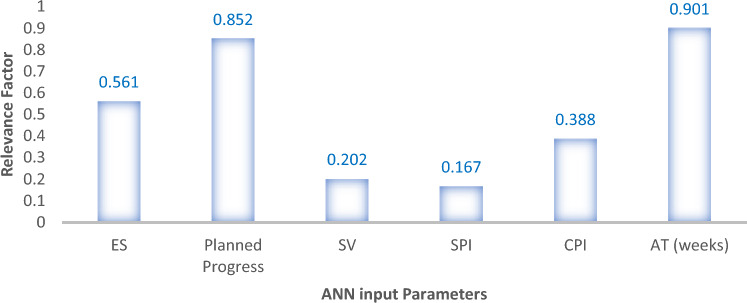
ANN model sensitivity analysis results.

**Figure 20:**
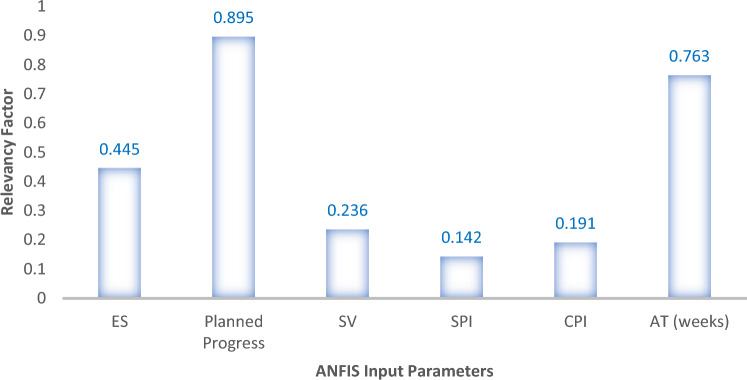
ANFIS model sensitivity analysis results.

Sensitivity analysis assesses the contribution of the individual independent variables to the output response (EV). For this purpose, the methods reported by Razavi et al.^80^ were adapted to determine which inputs had the greatest impact on the output variable. We used the relevancy factor (*r*), where r is in the range of [− 1, 1]. The r values were calculated using Eq. (11).

11 $$ r = \frac{{\sum\nolimits_{i = 1}^{n} {\left( {X_{k,i} - \overline{{X_{k} }} } \right)\left( {Y_{i} - \overline{Y} } \right)} }}{{\sqrt {\sum\nolimits_{i = 1}^{n} {\left( {X_{k,i} - \overline{{X_{k} }} } \right)^{2} \times \sum\nolimits_{i = 1}^{n} {\left( {Y_{i} - \overline{Y} } \right)^{2} } } } }} $$

where $X_{k,i}$ and $Y_{i}$ are the *i*th input and output, respectively; $\overline{Y}$ and $\overline{{X_{k} }}$ are the average values of the output and kth input, respectively; and n denotes the total number of data points. The computation results are presented in Figs. 19 and 20. From the plotted results, it can be observed that the major influencing parameters were the planned progress, actual time (AT) and earned schedule (ES) factors, with relevance scores of 0.895, 0.763 and 0.445, respectively. In contrast, the schedule variance (SV), schedule performance index (SPI) and cost performance index (CPI) factors had the minimum relevancy among the factors of 0.236, 0.142 and 0.191, respectively, for the ANFIS model sensitivity results. Similarly, for the ANN model, the actual time (AT) and planned progress were the maximum relevance factors, scoring 0.901 and 0.852, respectively, while the minimum relevance score of 0.167 was derived for the schedule performance index (SPI) factor. The computed sensitivity analysis results obtained are in agreement with the analytical findings of Zarei et al.^81^.

## Conclusions

The research assessment of the application of artificial intelligence in construction scheduling for efficient project management was achieved in this study with a two-story residential structure construction taken as a case study to design and evaluate the schedule and cost performance indicators. The following conclusions can be drawn:

- The construction project under study was executed by medium-sized firm with a planned duration of 95 days at an estimated direct cost of 25.8 million naira. The project performance indicators were evaluated through earned value analysis from 0–100% progress, at 5% increments, with a total of 17 tasks. This was carried out using Microsoft Project software, and data obtained from the computation were utilized for model development;
- Pearson’s correlation results obtained for the model variables indicated strong positive relationships between the response factor, earned value (EV), and the following performance indicators: planned progress, actual time (AT), earned schedule (ES), actual cost (AC) and cost variance (CV). Meanwhile, negative linear relationships were observed to exist for the schedule performance indicator and schedule variance factors;
- Data generated in this process were expertly selected for the input–output model variables’ formulation to improve project performance, reduce costs, and enhance overall project management. ANN and ANFIS were deployed for the smart modeling process using MATLAB software for the model simulation, training, testing and validation;
- The model’s prediction accuracy was evaluated using loss function parameters, namely the root mean squared error (RMSE) and mean absolute error (MAE). The results calculated indicated a better performance for the ANFIS model, with a MAE and RMSE of 1.9815 and 2.146, respectively, while ANN performed satisfactorily, with a MAE and RMSE of 2.257 and 2.4095, respectively. The model performance results showed it was adaptive and robust, dealing with complex relationships between the model variables to produce an accurate target response;
- The results suggest that these models can be effectively integrated into existing scheduling processes and have the potential to significantly improve project performance. The developed models also offer a viable and accurate means of providing project performance indicators that enable project/construction managers to proficiently monitor, control and execute projects with the designed quality, time and resources. Furthermore, details derived through this research study will contribute toward developing an essential template for efficient planning and accountability of construction projects, to prevent challenges such as cost overruns.

### Study’s limitations and recommendations

Investigative research on construction schedule evaluation using artificial intelligence tools is very important given that it can be applied to deal with non-linear complex problems better than conventional statistical approaches. The gains derived from this work will contribute essential information to the decision-making process in construction planning, monitoring and controlling, to achieve the optimum solution. The system datasets utilized for the smart intelligent modeling in this research study were, however, limited to two-story residential structure construction in the area of the study. Therefore, further investigation is recommended using different classes of buildings based on the intended use of the structure, along with the deployment of multiple hybrid AI algorithms such as the neural networks–genetic-fuzzy-logic hybrid algorithm.

## Supplementary Information

Supplementary Information.

## Data Availability

The datasets generated and/or analyzed during the current study are available from the corresponding author on reasonable request.

## Abbreviations

AT: Actual time
SV: Schedule variance
EV: Earned value
ES: Earned schedule
AC: Actual cost
SPI: Schedule performance indicator
CV: Cost variance
CPI: Performance indicator
ANN: Artificial neural networks
ANFIS: Adaptive neuro-fuzzy inference system
EVM: Earned value management
CPM: Critical path method
AI: Artificial intelligence
trimf: Triangular membership function
trapmf: Trapezoidal membership function
gbellmf: Generalized bell-shaped membership function
pimf: Pi-shaped membership function
guassmf: Gaussian membership function
gauss2mf: Gaussian combination membership function
psigmf: Product of two sigmoidal membership function
RMSE: Root mean squared error
MSE: Mean squared error
MAE: Mean-absolute-error
r^2^: Coefficient of determination
