# Supplementary material for: Application of neural networks and neuro-fuzzy models in construction scheduling
Source: Sci Rep. 2023 May 21;13:8199. doi: 10.1038/s41598-023-35445-5 (PMC10200797; doi:10.1038/s41598-023-35445-5)
Supplement: Supplementary file 1 — Supplementary Information. [file 41598_2023_35445_MOESM1_ESM.docx]

function [y1] = myNeuralNetworkFunction(x1)

%MYNEURALNETWORKFUNCTION neural network simulation function.

%

% Generated by Neural Network Toolbox function genFunction, 15-Sep-2021 03:20:14.

%

% [y1] = myNeuralNetworkFunction(x1) takes these arguments:

% x = 8xQ matrix, input #1

% and returns:

% y = 1xQ matrix, output #1

% where Q is the number of samples.

%#ok<*RPMT0>

% ===== NEURAL NETWORK CONSTANTS =====

% Input 1

x1_step1_xoffset = [2.22;5;-0.96;0.931;8240;1.012904034;2;638560];

x1_step1_gain = [0.0984251968503937;0.0210526315789474;1.52671755725191;11.1731843575419;3.07073436612366e-06;31.0595322713976;0.0930232558139535;2.12306111443724e-07];

x1_step1_ymin = -1;

% Layer 1

b1 = [2.0347143787808752;-1.4906007895534628;1.0405456148513699;0.56586324373695474;0.18818666973884796;-0.1769267001745258;-0.40154563919549541;1.0438492312149668;-1.4361329193693924;-1.8947706435524265];

IW1_1 = [-0.83627813577646104 -0.74414855207981245 -0.063637749099378457 -0.05795591610889058 -0.29795970543215938 -0.68761090869715924 0.43375337708858203 -0.8566742514072041;0.93428966240583877 -0.46744213411578528 -0.88541589772197626 0.56238024702880784 0.2882249755689687 -0.93101962981947506 -0.38850711883466427 -0.27930253788337883;-0.6474244560621274 -0.97429121562468568 0.71470065077373668 0.4571041862015166 0.54493131958527219 -0.9020845744122169 0.36081527281697268 -0.27027595921567532;-0.98384074732784432 -0.16902238611260603 -0.32722823792242339 0.69704911151583016 -0.99710077606105429 0.83210154847036144 0.5772799893770294 0.27996420284033041;-0.4823692495448284 0.6795311299964607 -0.56129209008482683 -0.51481418393350631 -0.92217972199542608 1.0542974907357001 0.21787185925160787 0.27527760544926833;-0.11299952947128804 0.48948279845635373 0.86877385161845633 0.64519844193921794 0.12332525525614632 0.55932048269120171 0.86050156901157226 0.98326566104951119;-0.71340920236314731 -0.77915493960077298 -0.91616976496867253 0.057894305510698692 -0.20885958153256184 -0.69037271038400683 -0.44994869116382047 -0.45498750810491906;0.83017094893351506 -0.84227359194342366 -0.85659183795405791 -0.12877498770942089 0.76908893803885015 0.38697467060967417 0.34625722569728173 -0.51864082577950943;-0.37471797154711384 0.28348863478997532 0.44246525822859112 -0.77366816070933941 0.48181214662207539 -0.56168899858013444 0.8366702533602054 -1.0059456674141989;-0.47560993133804813 0.87286591757028742 0.24563170486345365 0.63902712339350132 0.51095310403078265 -0.86814109295173358 0.79220570492467168 0.59241876417340733];

% Layer 2

b2 = -0.58360318344955797;

LW2_1 = [-0.37322884431871595 0.0027251499967432626 0.016844442206342645 -0.69521511992144847 -0.0069311550334863201 0.2202843681266563 -0.080019324696981212 0.10096545827479839 -0.66146222420908973 -0.31239181508385888];

% Output 1

y1_step1_ymin = -1;

y1_step1_gain = 1.98576800073871e-07;

y1_step1_xoffset = 646800;

% ===== SIMULATION ========

% Dimensions

Q = size(x1,2); % samples

% Input 1

xp1 = mapminmax_apply(x1,x1_step1_gain,x1_step1_xoffset,x1_step1_ymin);

% Layer 1

a1 = tansig_apply(repmat(b1,1,Q) + IW1_1*xp1);

% Layer 2

a2 = repmat(b2,1,Q) + LW2_1*a1;

% Output 1

y1 = mapminmax_reverse(a2,y1_step1_gain,y1_step1_xoffset,y1_step1_ymin);

end

% ===== MODULE FUNCTIONS ========

% Map Minimum and Maximum Input Processing Function

function y = mapminmax_apply(x,settings_gain,settings_xoffset,settings_ymin)

y = bsxfun(@minus,x,settings_xoffset);

y = bsxfun(@times,y,settings_gain);

y = bsxfun(@plus,y,settings_ymin);

end

% Sigmoid Symmetric Transfer Function

function a = tansig_apply(n)

a = 2 ./ (1 + exp(-2*n)) - 1;

end

% Map Minimum and Maximum Output Reverse-Processing Function

function x = mapminmax_reverse(y,settings_gain,settings_xoffset,settings_ymin)

x = bsxfun(@minus,y,settings_ymin);

x = bsxfun(@rdivide,x,settings_gain);

x = bsxfun(@plus,x,settings_xoffset);

end
